# Epidemiology and Predictors of End-stage Renal Disease in Taiwanese Children With Idiopathic Nephrotic Syndrome

**DOI:** 10.2188/jea.JE20120033

**Published:** 2012-11-05

**Authors:** Jei-Wen Chang, Hsin-Lin Tsai, Ling-Yu Yang, Tzeng-Ji Chen

**Affiliations:** 1 Division of Pediatrics, Department of Pediatrics, Taipei Veterans General Hospital, Taipei, Taiwan; 2 Division of Pediatric Surgery, Department of Surgery, Taipei Veterans General Hospital, Taipei, Taiwan; 3 Department of Family Medicine, Taipei Veterans General Hospital, Taipei, Taiwan; 4 Institute of Clinical Medicine, National Yang-Ming University, School of Medicine, Taipei, Taiwan

**Keywords:** idiopathic nephrotic syndrome, long-term outcome, children, end-stage renal disease

## Abstract

**Background:**

The incidence of idiopathic nephrotic syndrome (INS) varies among countries, with Asia reporting a higher incidence in comparison with Western countries. We investigated the epidemiologic features of INS and attempted to identify factors that predispose individuals to develop end-stage renal disease (ESRD).

**Methods:**

Claims data from the Taiwanese National Health Insurance program from 1996 to 2008 were used to investigate the epidemiologic features and clinical variables of INS (International Classification of Diseases, Ninth Revision, Clinical Modification code, 581) in children younger than 18 years.

**Results:**

We enrolled 4083 children (male-female ratio, 1.91:1). During the 13 years of observation, annual incidence decreased from 9.91 to 3.36 per 100 000 children. Annual number of hospital admissions progressively decreased during the first 3 years after diagnosis. At 3.14 ± 2.77 years after INS onset, ESRD had developed in 145 (3.6%) children. Independent predictors of ESRD included older age at onset, acute renal failure (ARF), hypertensive encephalopathy, and a histologic subtype with focal segmental glomerulosclerosis (FSGS).

**Conclusions:**

Pediatric INS in Taiwan was more frequent in boys. Unlike India, the current incidence of pediatric INS in Taiwan is very similar to that reported in Western studies. Older age at disease onset, ARF, hypertensive encephalopathy, and FSGS on biopsy are important predictors of poor renal outcome.

## INTRODUCTION

Idiopathic nephrotic syndrome (INS) is characterized by edema, massive proteinuria, hypoalbuminemia, and hyperlipidemia. INS most commonly appears in children aged from 4 to 8 years.^1^ As many as 80% to 90% of children with INS respond to the steroid protocol developed by the International Study of Kidney Disease in Children. However, 60% to 80% of steroid-responsive nephrotic children will relapse, and about 60% will relapse more than once, some as many as 5 times. The frequency of relapse progressively decreases after adolescence. Although long-term outcomes are generally favorable,^1^ children with steroid-resistant INS may progress to end-stage renal disease (ESRD).

The reported annual incidence of INS is 2 to 16.9 cases per 100 000 children.^2^^–^^5^ Many studies have suggested that ethnicity has a role in the epidemiology of INS^6^: the incidence of INS is markedly higher in South Asian children than in Western children, for example.^2^^–^^5^^,^^7^ During the first INS episode, hospital admission is always necessary in Taiwan, to allow for laboratory studies, albumin and diuretic therapy, education, and for social reasons. After the first INS episode, children are not admitted to hospital unless they relapse with gross edema, develop complications of nephrotic syndrome, or receive pulse therapy or aggressive immunosuppressive therapy for steroid resistance. A greater number of hospital admissions indicates a more cases of relapsing–progressive INS. To our knowledge, no nationwide study of INS incidence has been conducted in Taiwanese children. In Taiwan, the coverage rate of the National Health Insurance (NHI) system—a compulsory social health care system established in 1995—had reached 98% at the end of 2006.^8^ Thus, we could use data collected from this database to investigate INS incidence, the number of recorded hospital admissions, and predictors of progression to ESRD in pediatric INS.

## METHODS

### Data sources

The NHI database is a large computerized database that includes all medical claims recorded in the Taiwanese NHI program, a single-payer payment system created in 1995. After de-identification and encryption, the research databases are released for medical research. Diagnoses in the NHI research databases are coded using the International Classification of Diseases, Ninth Revision, Clinical Modification (ICD-9-CM) coding scheme. The Bureau of NHI cross-checks and validates medical charts to ensure the accuracy of diagnosis coding in the NHI database. When fraudulent coding, discrepancies, overcharging, or malpractice are discovered, physicians are subject to penalties or suspension. Therefore, the fidelity of coding in the database is considered to be high. To protect privacy, each patient in the NHI database is assigned a unique identification number that cannot be traced back to the individual patient. The date of the first visit is recorded as the date of disease onset. Multiple hospitalization discharge claims with the same personal identification number were counted once, so that INS incidence was accurately calculated.

### Study population

Using ICD-9-CM codes in the NHI database, we identified all first-time admissions from 1996 through 2008 of patients aged between 6 months to 18 years with INS (ICD-9-CM code 581) as the principal or secondary diagnosis. We excluded children with secondary nephrotic syndrome (ICD-9-CM code 581.81). Demographic data for the child population of the Taiwan area were obtained from the Department of Statistics, Ministry of the Interior, Republic of China.^9^

### Variables

We obtained information on age at presentation, sex, date of first hospital admission, month and year of admission, number of readmissions in the first, second, and third years after initial presentation, and total number of admissions. Coexisting diagnoses of respiratory infection (ICD-9-CM codes 460–466, 480–487) at the time of initial presentation, atopy history during the study period (ICD-9-CM codes 477, 493, 691), and complications such as acute renal failure (ARF; ICD-9-CM code 584), spontaneous bacterial peritonitis (ICD-9-CM code 567), cellulitis (ICD-9-CM code 680–682), urinary tract infection (UTI; ICD-9-CM code 599.0), hypertensive encephalopathy (ICD-9-CM code 437.2), and thromboembolism episode (ICD-9-CM code 451, 453, 415, 415.1) were also obtained to identify comorbidities and complication rates.

### Renal outcomes

Renal failure was defined based on the presence of ESRD (ICD-9-CM code 585). To evaluate long-term renal outcomes, we calculated time from disease onset to ESRD occurrence.

### Statistical analysis

First, we calculated annual incidence of INS from 1996 to 2008 by dividing the number of INS hospitalizations per 100 000 children of the same age between 1996 and 2008. Identified patients were divided into those who developed ESRD (group 1) and a control group (group 2) of patients who did not develop ESRD. Descriptive statistics were used to analyze demographic data and the distributions of each variable among the study population. Continuous data were described as mean plus SD. Categorical variables were expressed as percentages. We used the chi-square trend test (linear-by-linear association) to examine temporal (yearly) trends in INS incidence. Renal survival was analyzed by the Kaplan–Meier method. For the analysis of risk factors of progression to ESRD, univariate analysis was performed using clinical variables, including sex, age, number of admissions, and complications, for groups 1 and 2. The same factors were included in multivariate analysis, regardless of their significance in univariate analysis. Multivariate analysis of independent factors regarding progression to ESRD was performed using the Cox proportional hazards model. All procedures were done using SPSS, version 16.0. A *P* value less than 0.05 was considered to indicate statistical significance.

## RESULTS

### Demography, characteristics of patients, and epidemiologic features

**Figure 1.**
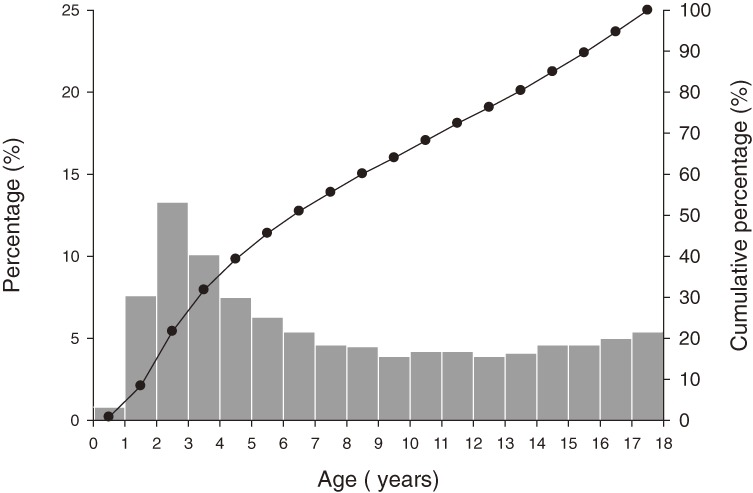
Age distribution and cumulative percentage of idiopathic nephrotic syndrome in Taiwanese children.

**Figure 2.**
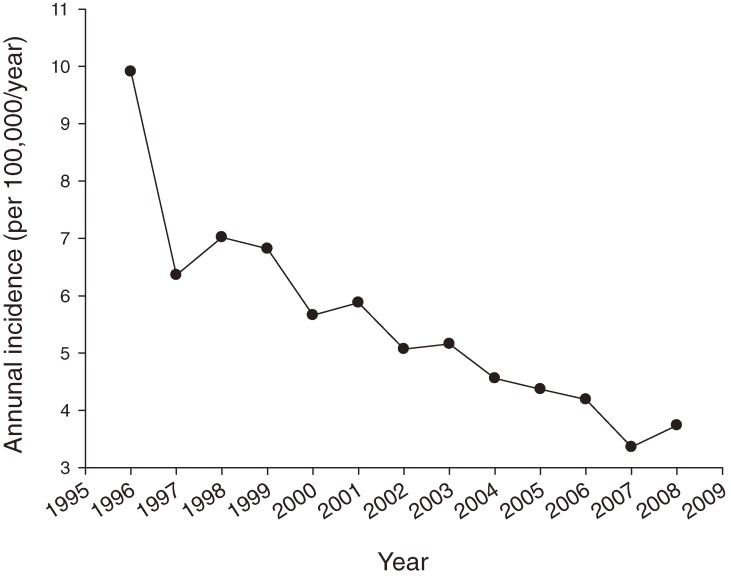
Annual incidence of admission for idiopathic nephrotic syndrome (INS) among Taiwanese children younger than 18 years. Annual incidence of INS decreased significantly from 1996 through 2008 (*P* = 0.002, linear-by-linear association).

A total of 4083 children and adolescents aged from 6 months to 18 years were hospitalized for INS during the 13-year study period. The number of males was 2680, and the male-female ratio was 1.91:1. The age distribution is shown in Figure 1. The average age of patients at initial diagnosis was 8.06 ± 5.20 years. INS incidence varied among age groups, and the highest incidence was among children aged 2 to 3 years. Annual incidence ranged from 9.91 to 3.36 per 100 000 children per year (Figure 2). The average incidence rate was 5.66 per 100 000 children per year. There was a significant decrease in the annual incidence of INS in children during our study period, 1996 to 2008 (*P* = 0.002, linear-by-linear association).

### Seasonal distribution

**Figure 3.**
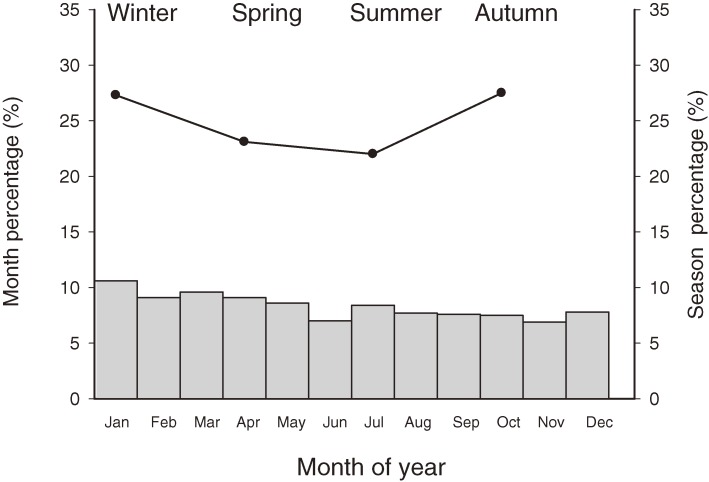
Monthly and seasonal distribution of the incidence of idiopathic nephrotic syndrome. The highest incidence was in winter and spring.

The monthly and seasonal distribution of patients, as shown in Figure 3, was 27.3% in spring (March to May), 23.1% in summer (June to August), 22.0% in autumn (September to November), and 27.5% in winter (December to February). Although there was no statistically significant difference in incidence among seasons (*P* = 0.059, analysis of variance test), INS was more common in winter and spring than in autumn (*P* = 0.03 for both comparisons; least significant difference post-hoc test).

### Renal outcome and factors associated with progression to ESRD

**Figure 4.**
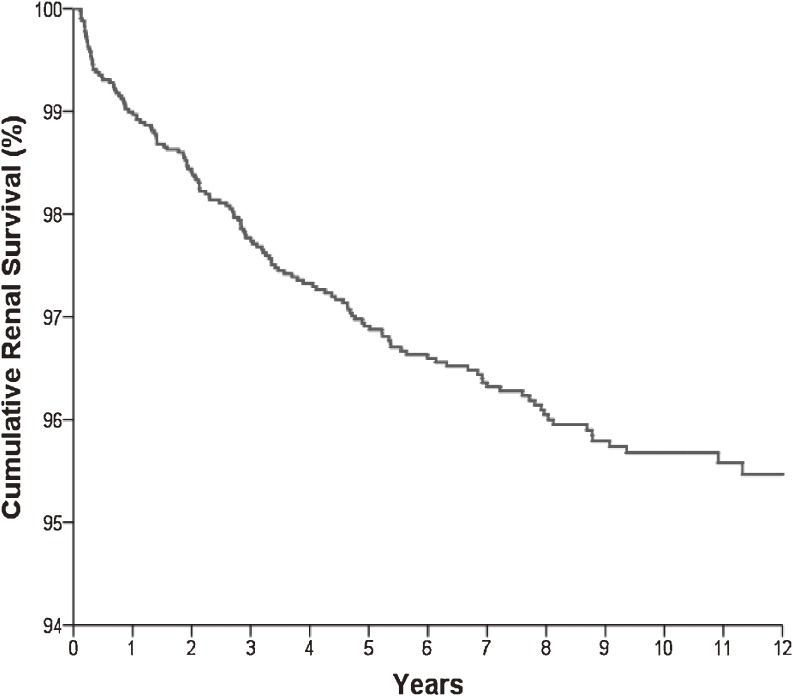
Cumulative renal survival in patients with idiopathic nephrotic syndrome. Renal survival was 96.9% at 5 years and 95.7% at 10 years.

During a mean follow-up of 7.70 ± 3.81 years, 145 (3.6%) of the 4083 claims showed progression to ESRD. Average time from onset of INS to ESRD was 3.14 ± 2.77 years. Renal survival was 96.9% at 5 years and 95.7% at 10 years (Figure 4). Focal segmental glomerulosclerosis (FSGS) accounted for 17.9% of progression to ESRD.

Table 1 shows a comparative summary of clinical and biochemical characteristics in groups 1 and 2. The overall complication rate was 14.5% among patients in group 1 and 10.1% among those in group 2. Although the complication rate was higher in group 1, the difference was not statistically significant. The most frequent complication was UTI: 7 (4.8%) and 259 (6.6%) episodes were noted in groups 1 and 2, respectively. The annual number of hospital admissions progressively decreased after the initial admission. Group 1 was significantly older at initial presentation and had higher rates of ARF and hypertensive encephalopathy in univariate analysis. The average annual hospitalization rate in the first, second, and third years of disease, total number of admissions during follow-up, and proportion of patients with FSGS were also significantly higher in group 1 than in group 2.

**Table 1.** Comparison of demographic and clinical characteristics of patients who developed end-stage renal disease (group 1) and those who did not (group 2)

	Group 1	Group 2	*P* value
No. of patients	145	3938	
Male/female	89/56	2591/1347	0.27
Age (years)	12.00 ± 4.83	7.91 ± 5.16	<0.001*
Respiratory tract infection	17 (11.7%)	868 (22.0%)	0.003*
Atopy history	18 (12.4%)	450 (11.4%)	0.71
Complication	21 (14.5%)	399 (10.1%)	0.09
ARF	6 (4.1%)	44 (1.1%)	0.001*
SBP	2 (1.4%)	42 (1.1%)	0.67
Cellulitis	3 (2.1%)	42 (1.1%)	0.21
UTI	7 (4.8%)	259 (6.6%)	0.40
Hypertensive encephalopathy	3 (2.1%)	2 (0.05%)	0.001*
Thromboembolism	0 (0%)	10 (0.25%)	1
Histologic subtype			
Focal and segmental glomerulosclerosis	26 (17.9%)	103 (2.6%)	<0.001*
Unspecified or other pathologic lesion	119 (82.1%)	3835 (97.4%)	
Yearly number of admissions			
1st year	1.62 ± 3.31	0.80 ± 1.58	0.003*
2nd year	0.72 ± 1.89	0.30 ± 1.00	0.010*
3rd year	0.53 ± 1.30	0.18 ± 0.83	0.002*
Total admissions during follow-up	3.86 ± 6.03	1.81 ± 4.26	<0.001*
Time of follow-up	8.29 ± 3.91	7.68 ± 3.80	0.06

*P < 0.05 in univariate analysis.

ARF, acute renal failure; SBP, spontaneous bacterial peritonitis; UTI, urinary tract infection.

In multivariate analysis (Table 2), factors that independently predicted progression to ESRD were older age at initial presentation (b = 0.15, *P* < 0.001, hazard ratio = 1.16; 95% CI = 1.12, 1.21), ARF (b = 0.97, *P* = 0.038, hazard ratio = 2.64; 95% CI = 1.06, 6.61), hypertensive encephalopathy (b = 4.99, *P* < 0.001, hazard ratio = 146.23; 95% CI = 12.72, 1680.71), and presence of FSGS (b = 1.58, *P* < 0.001, hazard ratio = 4.87; 95% CI = 2.92, 8.13).

**Table 2.** Associations of clinical variables with progression to end-stage renal disease in Cox proportional hazards analysis

Predictor Variables	HR	LCL	UCL	*P*
Age	1.16	1.12	1.21	<0.001
ARF	2.64	1.06	6.61	0.038
Hypertensive encephalopathy	146.23	12.72	1680.71	<0.001
Focal and segmental glomerulosclerosis	4.87	2.92	8.13	<0.001

ARF, acute renal failure; HR, hazard ratio; LCL, lower 95% confidence interval; UCL, upper 95% confidence interval.

## DISCUSSION

This was the first nationwide population-based study of childhood INS in Taiwan, which has an estimated population of 23 million. The compulsory NHI program, launched on 1 March 1995, provides health care to all Taiwanese citizens and covered 98% of the total population by 2006. According to official government statistics, 98% of Taiwanese are ethnic Chinese and 2% are aboriginal Taiwanese. Therefore, this computerized database is a good source of data for a study of INS among an ethnically homogeneous population.

Recent epidemiologic studies show that INS incidence and prognosis among children differ by ethnicity, race, and geographic area. New Zealand had a reported incidence of 1.9 per 100 000 children younger than 15 years in 2007.^2^ In the United States, annual incidence was also 1.9 per 100 000 in white children and 2.8 per 100 000 in non-white children.^3^ A UK study found that the annual incidence of INS was 2.6 per 100 000 children of European ancestry and 3.4 among those with Afro-Caribbean ancestry.^5^ INS incidence is reported to be higher in Asia than in Western Countries,^2^^–^^5^^,^^7^ and India has the highest annual incidence (16.9 per 100 000 children).^7^ Our data show a clear decreasing trend in INS incidence among Taiwanese children during recent years. The reasons for this decrease in incidence are unknown. However, it is known that poverty increases the risks of infection, and studies have noted higher INS incidence in groups with low socioeconomic status.^3^ From 1996 to 2008, the average gross domestic product in Taiwan rose from US$13 428 in 1996 to US$17 399,^10^ and the decrease in INS incidence may be related to improvements in socioeconomic status and environmental sanitation. Our study is consistent with epidemiologic findings in the literature on INS, which show a peak age of INS onset of 2 to 3 years, male predominance,^11^ and seasonal incidence peaks in spring and winter.

Children with INS have a higher risk of infection, the most common of which is upper respiratory tract infection, followed by UTI, peritonitis, and pneumonia.^12^ Among infection-related complications, UTI (6.51%) was the most common in our study. The reported rate of peritonitis is 2% to 15.8%^12^^,^^13^; however, the rate was lower (1.08%) in the present study. ARF is an uncommon complication of INS in children.^14^ Possible causes of ARF include bilateral renal vein thrombosis, interstitial edema, tubular obstruction, rapid progression of the original glomerular disease, and acute tubular necrosis secondary to sepsis or hypovolemia. ARF is reversible, and recovery of renal function can be expected in most patients.^14^^,^^15^ Risk factors for ARF include severe hypoalbuminemia, older age (in adults), and infection (in children).^16^ The incidence of ARF in this study was 1.22%, which is consonant with the rates of 0.8% to 5% noted in previous reports.^17^ Another very rare complication, acute hypertensive encephalopathy, is a transient neurologic dysfunction induced by marked increases in blood pressure during a moderate to severe nephrotic state.^18^ Clinical presentation includes mental change, seizures, headache, and visual disturbance. Hypertensive encephalopathy was found in 0.12% of our patients. INS is associated with a hypercoagulable state and thrombosis due to urinary loss of anticoagulants such as antithrombin, elevated plasma fibrinogen concentration, increased platelet aggregability, diuretic use, corticosteroid treatment, venepuncture, and immobility. Thromboembolism occurs in 2% to 5% of children with nephrotic syndrome.^19^ However, the rate of thromboembolism was only 0.24% in our study, possibly because thromboembolism is, in general, less common in Asian populations.^20^ In total, 420 episodes of complications were identified in the 4083 cases, which corresponds to an overall complication rate of 10.29%. ARF, hypertensive encephalopathy, and thromboembolism occurred in 4.1% vs 1.1%, 2.1% vs 0.05%, and 0% vs 0.25% of the patients in groups 1 and 2, respectively. ARF and hypertensive encephalopathy were independent variables associated with progression to ESRD.

Causes of INS include minimal change disease (MCD), mesangial proliferation, and FSGS. The standard treatment for INS is steroid treatment, and 93% of patients with histologic findings of MCD respond to treatment with prednisone.^1^ Newly diagnosed patients are always admitted for diagnostic, supportive care with diuretics and albumin and also for serious complications such as pulmonary edema, pleural effusion, and bacterial infection. Most patients will have 1 or 2 relapses (++ or more on albustix for ≥3 consecutive days). Early relapse after onset and a short remission period just before a recent relapse are independent risk factors for subsequent relapse.^21^ Spontaneous remission of relapse occurs in 23% of frequent relapsers and in 10% of steroid-dependent patients between day 4 and day 14 after onset of relapse.^22^ Therefore, in uncomplicated cases of relapsed INS, hospital admission is not always required. Relapses decrease as the child gets older: 50% to 70% of children are relapse-free at 5 years of illness^23^ and 58% to 90% are relapse-free after age 18 years.^24^^,^^25^ For steroid-resistant INS, the clinical course is typically more complex due to refractory edema, severe infections, thromboembolic complications, and refractory hypertension, which result in more hospital admissions. The re-admission rate among our patients was high: 49% were re-admitted at least once and about 15% were admitted more than 3 times during the observation period. The average total number of annual admissions in the first, second, and third years was 0.83, 0.32, and 0.19, respectively, which is lower than the relapse rate reported in the literature. This study provides evidence that children with INS have a high re-admission rate, which generally declines with the frequency of relapse as children age.

Although INS can affect any age group, age at initial presentation has an important impact on disease distribution and steroid response.^26^ The younger the child at onset, the greater the likelihood that the lesion is MCD. If onset occurs before age 5 years, the likelihood of MCD is greater than 90%. Onset after adolescence decreases the risk to 20% to 30% but increases the risk of FSGS to 30%.^11^ Previous research found that the older the patient at onset, the higher the frequency of steroid resistance and the worse the prognosis.^27^ We also found a statistically significant relationship between older age at onset and progression to ESRD.

Renal histology is also an important predictor of long-term outcome. FSGS is associated with worse outcomes: only 20% to 30% of children with FSGS respond to steroids, and 25% to 62% of children with FSGS develop ESRD within 5 to 10 years.^28^ It is difficult to compare the present data on long-term outcomes of children with INS with those obtained from other studies, as most of those studies enrolled patients who had steroid-resistant INS or FSGS. Cohort studies have reported that renal survival at 5 and 10 years was 75% to 92% and 50% to 86%, respectively, in patients with steroid-resistant nephrotic syndrome.^28^^–^^30^

To the best of our knowledge, this is the largest study to investigate the epidemiology and prognostic factors of progression to ESRD in Taiwanese children with INS. However, the study had several limitations. First, there were no available data on response to steroid treatment, which could be of prognostic utility. Another limitation was the lack of data on serology and remission induction with second-line agents in patients with steroid resistance.

In summary, this study yielded several noteworthy findings. Pediatric INS in Taiwan occurs more frequently in boys. Over the 13-year period of study, the annual incidence of INS in children decreased from 9.91 to 3.36 per 100 000. In contrast to India and other Asian countries, the current incidence of pediatric INS in Taiwan is very similar to that in Western countries. The most frequent complication was UTI. The long-term renal prognosis of INS is positive, as only a small number of patients progressed to ESRD. Older age at disease onset, development of ARF, hypertensive encephalopathy, and FSGS are risk factors for progression to ESRD.
